# Comprehensive Conservative Management as Rescue Therapy After Haemodialysis Failure: Two Case Reports

**DOI:** 10.3390/clinpract16020025

**Published:** 2026-01-25

**Authors:** Francesca K. Martino, Alessandro Martella, Francesca Fioretti, Leda Cattarin, Federica L. Stefanelli, Federico Nalesso

**Affiliations:** Nephrology, Dialysis and Transplantation Unit, Department of Medicine (DIMED), University of Padova, 35128 Padua, Italy; alessandro.martella.1@studenti.unipd.it (A.M.); francesca.fioretti.2@studenti.unipd.it (F.F.); leda.cattarin@aopd.veneto.it (L.C.); luciafederica.stefanelli@unipd.it (F.L.S.); federico.nalesso@unipd.it (F.N.)

**Keywords:** conservative management, hemodialysis, end-stage kidney disease, frailty, case-report

## Abstract

**Background:** Comprehensive conservative management (CCM) is a possible option in end-stage clinical disease, requiring multidisciplinary support and offering survival comparable to dialysis while improving quality of life in frail patients. Despite its potential benefits, CCM is often underutilized because nephrologists may perceive it as less effective compared to dialysis. We present two case reports of hemodialysis failure and of successful CCM. **Case presentation:** We present two case reports of elderly female patients—referred to as Patient 1 and Patient 2—who had multiple comorbidities but preserved urine output. Both patients, in accordance with their medical team, chose to discontinue hemodialysis due to poor treatment tolerance and declining overall health. They were successfully managed with CCM, leading to follow-up that revealed survival beyond 24 months, improvements in metabolic complications and quality of life, and a reduction in hospitalizations. **Conclusions:** These case reports demonstrate the effectiveness of dietary and medical management for end-stage kidney disease, particularly when dialysis negatively affects patients’ clinical conditions and quality of life. They also highlight the importance of considering CCM as a preferable option for frail elderly patients facing kidney failure.

## 1. Introduction

Population aging in the twenty-first century has resulted in a growing number of elderly patients with end-stage kidney disease (ESKD) in Western countries. The US Renal Data System reported an ESKD incidence of 1447 per million among individuals aged 75 years or older, nearly three times that in individuals aged 45 to 64 years [1]. Similarly, the 2023 European registry of kidney replacement therapy documented a prevalence of 2803 per million in patients aged 75 or older, approximately double that observed in patients aged 45 to 65 years [2]. Older ESKD patients are more likely to present with a higher comorbidity index, including diabetes, hypertension, and cardiovascular disease [3,4,5]. Among patients over 80 years of age with an estimated glomerular filtration rate (eGFR) below 15 mL/min/1.73 m^2^, life expectancy is estimated to be less than three years, regardless of chronic kidney disease treatment [6]. Cardiovascular death occurs in approximately 60% of ESKD cases [6,7], likely due to the elevated cardiovascular comorbidity index in this population [8,9,10].

Despite advances in general care and technical procedures, dialysis does not consistently improve survival in elderly patients with high comorbidity. As early as 1996, Soucie et al. reported a significant increase in mortality within the first 100 days of dialysis for individuals over 75 years, with a fivefold higher risk of death compared to ESKD patients aged 45 to 65 years [11]. Subsequent studies have confirmed that mortality risk in ESKD patients increases with age and comorbidity index [12,13,14]. Additionally, mortality risk in this population is influenced by socio-economic factors, including social isolation, family context, type of care, and the broader health care system [15,16]. In frail ESKD patients, initiation of dialysis is associated with a substantial decline in functional status, further loss of autonomy [17,18,19], and diminished quality of life [20,21,22,23].

Within this context, comprehensive conservative management (CCM) may offer significant clinical benefits [24,25]. CCM encompasses dietary interventions, strategies to slow the progression of kidney disease, and pharmacological treatments to address disease-related abnormalities, including metabolic acidosis, calcium and phosphate imbalances, anemia, and electrolyte disturbances. Dietary management primarily involves a low-protein diet with reduced intake of sodium, phosphorus, and potassium [26]. Personalization of dietary interventions should account for comorbidities and individual patient needs. Although protein restriction can compromise nutritional status in patients at risk of protein-energy wasting and sarcopenia [27,28,29,30,31], these risks are mitigated by ensuring adequate energy intake (25–35 kcal/kg body weight per day) and avoiding excessive protein reduction to below 0.55–0.6 g/kg body weight per day. Maintaining sufficient caloric intake is essential to prevent protein catabolism and preserve lean body mass. Further reduction in protein intake to 0.3–0.4 g/kg body weight daily necessitates supplementation with keto-analogues to provide essential amino acids [26,32,33,34,35]. For mineral requirements, the KDOQI “Clinical Practice Guideline for Nutrition in CKD: 2020 Update” does not specify exact targets but recommends adjusting potassium and phosphorus intake to maintain normal serum levels [26]. Sodium intake should be limited to less than 100 mmol per day, equivalent to 2.3 g per day. A surveillance and educational program to improve adherence to dietary and pharmaceutical regimens could be key to preventing nutritional impairment [35,36].

Although evidence supports the clinical benefits of CCM in frail and elderly ESKD patients, nephrologists often do not consider CCM a standard treatment option for this population [24,25,37]. For older individuals with ESKD who are unfamiliar with available therapies, treatment decisions are largely influenced by the information provided by their nephrologist, whose perspectives can significantly shape patient choices. Discussing treatment options remains challenging for nephrologists and other clinicians [38,39,40,41]. Clinicians are expected to guide patients despite limited evidence regarding the comparative benefits of dialysis and CCM. They must also address the widespread perception that dialysis is always necessary and life-sustaining. Given potential cognitive impairment in this patient group, clinicians should support choices that reflect patient preferences. Additionally, they must balance clinical needs with social and economic considerations, working collaboratively with caregivers.

### Perspectives for Clinical and Assistive Practice

CCM is frequently viewed as a marginal therapy reserved for patients lacking further treatment options, whereas dialysis is often considered the sole life-sustaining intervention. However, these case reports challenge this perception by demonstrating that CCM can be effective even in patients who do not benefit clinically from hemodialysis. Personalized medicine requires offering the most appropriate treatment based on the patient’s condition, preferences, and current evidence. Nephrologists and physicians should increasingly consider CCM as a therapeutic option, particularly given the rising number of frail and elderly patients with ESKD.

## 2. Case Presentation

All patient conditions and treatments are described in accordance with the CARE case report guidelines.

### 2.1. Patient 1

Patient 1, a woman, attended a nephrology outpatient clinic from 2016 following a diagnosis of mesangium-proliferative glomerulonephritis and subsequent development of nephrotic proteinuria with rapid decline in kidney function despite treatment. Her medical history included hypertensive cardiomyopathy, bilateral hip osteoarthritis, previous left femoral fractures treated with prosthesis replacement, chronic gastritis, and anxious-depressive syndrome. In 2017, she required renal replacement therapy, initiated as hemodialysis via a native arteriovenous fistula twice weekly, considering her refusal of the peritoneal dialysis program. This approach effectively managed chronic kidney disease (CKD) without a significant reduction in urine output or dialysis-related complications. From 2017 to October 2022, she underwent two 4-h hemodialysis sessions per week. The dialysis prescription included a blood flow rate of 300 mL/min, a dialysate flow rate of 500 mL/min, a polysulfone high-flux dialyzer with a surface area of 1.8 m^2^, and dialysate containing sodium 140 mmol/L, potassium 3 mmol/L, calcium 1.5 mmol/L, and bicarbonate 32 mmol/L. Ultrafiltration was set at 5–7 mL/kg per hour only when predialysis weight exceeded dry weight. Anticoagulation consisted of a 500 IU heparin bolus followed by 500 IU per hour, with heparin discontinued during the final hour of dialysis. Table 1 presents her blood test results and clinical indices during dialysis.

In June 2022, patient 1 developed bacterial endocarditis caused by oxacillin-resistant Staphylococcus aureus, which required prolonged hospitalization and aortic valve replacement surgery. Postoperatively, she developed atrial fibrillation. After 51 days of hospitalization, in late October 2022, she requested discontinuation of hemodialysis, and she declined the peritoneal dialysis option. Given her determination and preserved urine output of 1–1.2 L per day, the nephrology team reduced dialysis frequency to once weekly. In December 2022, she was rehospitalized for 11 days due to pneumonia with respiratory distress, requiring continuous positive airway pressure (CPAP) therapy and intravenous antibiotics.

**Figure 1:**
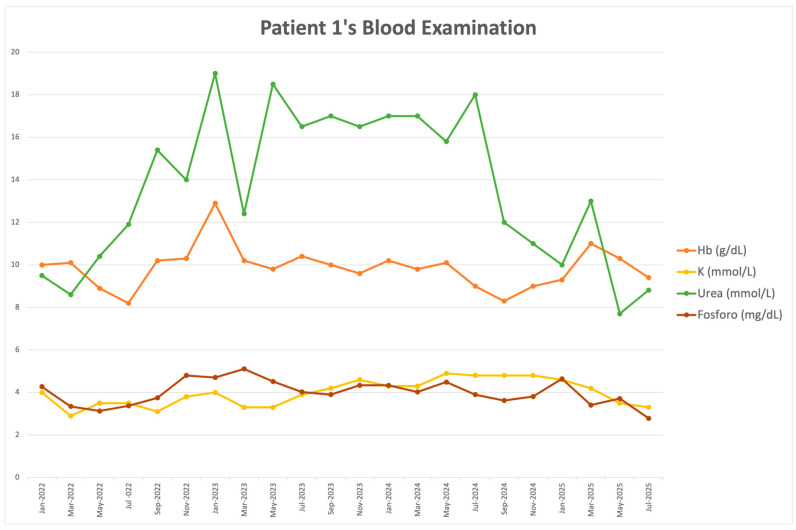
Patient 1’s levels of hemoglobin (red line), potassium (yellow line), phosphate (brown line), and urea (green line) before and after CCM. 2 HD/w and 1 HD/w correspond to 2 hemodialysis sessions and one hemodialysis session per week, respectively. During CCM, the patient received a very low-protein diet (0.5 g/kg/d) and 10 keto-analogue tablets per day. H corresponds to the hospitalization period.

In January 2023, she was referred to the CCM outpatient clinic for evaluations scheduled every six weeks. At that time, she weighed 51 kg, was 81 years old, and was entirely dependent on a caregiver. Furthermore, she had a moderate impairment in ambulation and required a wheelchair for mobility outside the home due to limited physical capacity. A low-protein diet was prescribed, providing 26 g of protein and 1600 kcal daily, with approximately 400 milligrams of phosphate and 5 g of sodium per day, along with full-dose keto-analogues. Her condition improved, as evidenced by increased appetite and stabilization of weight and nutritional parameters. Nephrology follow-up was discontinued in June 2025 after 29 months of CCM due to her death following a new episode of endocarditis, which was associated with worsening heart and kidney failure. During this acute episode, she refused renal replacement therapy. Blood tests and clinical features before and after initiation of the very low-protein diet are summarized in Table 1. Notably, her kidney function was severely impaired, with an average creatinine clearance of 4 mL/min, as expected in dialysis patients. Figure 1 highlights periods of twice-weekly and once-weekly hemodialysis, as well as 3 months and 1 year after CCM initiation. After 1 year, both weight and pharmacological treatment remained stable, with no changes in diuretics, phosphate binders, or other medications.

### 2.2. Patient 2

Patient 2 was a 79-year-old woman with multiple comorbidities, including diabetes, hypertension, ischemic heart disease, peripheral artery disease, and a history of rectal cancer. She was referred to nephrology care for ESKD in November 2020 and subsequently enrolled in the predialysis program. Previous abdominal surgery precluded peritoneal dialysis, so an arteriovenous fistula was created in early 2021. Hemodialysis was initiated in early 2022 due to worsening symptoms, including nausea, vomiting, dyspnoea, and chest pain. She underwent three-hour sessions thrice weekly. During the final three months of dialysis, she transitioned from standard hemodialysis to acetate-free biofiltration with a blood flow of 300 mL/min, dialysate flow of 500 mL/min, an AN69 dialyzer, with a dialysate conductivity of 15 ms/cm, sodium bicarbonate (145 mmol/L) reinfusion rate of 2.5 L per hour, and anticoagulation with Enoxaparin 2000 IU. Despite this change, she experienced symptomatic hypotension, profound asthenia, and significant weight loss, negatively affecting her quality of life. In August 2022, she was readmitted to the nephrology ward due to worsening general condition and an episode of atrial fibrillation during a hemodialysis session. Given her residual kidney function (creatinine clearance approximately 6 mL/min) and her preferences, the care team discontinued dialysis and initiated a very low-protein diet supplemented with keto-analogues. At that time, she weighed 46 kg, was 79 years old, and was entirely dependent on a caregiver for activities of daily living. Specifically, she required caregiver assistance for mobility in all contexts due to instability during ambulation.

**Figure 2:**
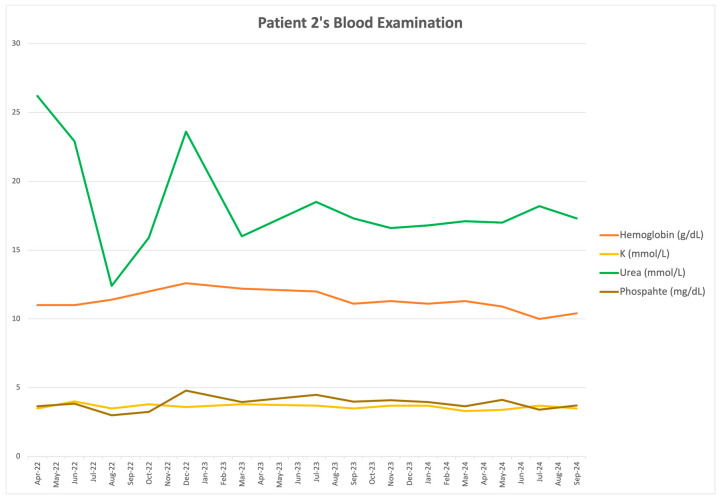
Patient 2’s levels of hemoglobin (red line), potassium (yellow line), phosphate (brown line), and urea (green line) before and after CCM. VLPD very low protein diet (0.4 g/kg/d) supported by 10 keto-analogues tablets per day, low protein diet (0.5-0.6 g/kg/d) supported by seven keto-analogues tablets per day. H corresponds to the hospitalization period.

The CCM program utilizes blood examinations to assess kidney function, anemia, calcium and phosphate metabolism, sodium and potassium balance, metabolic acidosis, uric acid control, and malnutrition indices. Consultations with a nephrologist occurred every 4–8 weeks, depending on clinical status. A very low-protein diet was prescribed, providing approximately 0.4 g of protein and 30 Kcal/kg of body weight per day, with 350 mg of phosphate and 3.5 g of sodium per day, supported by full doses of keto-analogues and essential amino acids. After five months, patient 2 exhibited an unexpected increase in uremic toxins, attributed to poor compliance with the very low-protein diet. In collaboration with the dietitian, daily protein intake was increased to 0.5–0.6 g/kg of body weight to allow a less restrictive and more palatable diet, providing approximately 25 Kcal/kg of body weight per day, 500 mg of phosphate, and 3.5 g of sodium. Supplementation with keto-analogues was reduced to 30% of the full dose. In subsequent months, the patient maintained good compliance with the low-protein diet and gained weight to 53 kg, without deterioration in malnutrition indices or impairment of glycemic control. The MUST score indicated a low risk of malnutrition, and albumin and prealbumin levels remained stable within the normal laboratory range. Patient 2’s general condition improved, given her age and frailty. Nephrology follow-up was discontinued in September 2024, after 25 months of comprehensive management, due to the patient’s sudden death. Blood test results and other clinical parameters are presented in Table 2, organized by treatment period. During the CCM period, patient 2 achieved a good phosphate balance, allowing discontinuation of phosphate binders and a reduction in the erythropoietin dose. The analysis focused on periods before dialysis discontinuation, 3 months after CCM initiation, and 1 year after CCM initiation. Figure 2 illustrates changes in key blood test results, treatment type, dietary recommendations, and hospitalization duration throughout the follow-up period.

## 3. Discussion

CCM has traditionally been reserved for frail and elderly patients, in whom nephrologists anticipate mortality within the medium term, or as an initial step before renal replacement therapy. These two cases were presented to illustrate the potential role of CCM as a rescue therapy in patients with ESKD who are intolerant of hemodialysis but maintain preserved urine output.

Although there is growing evidence supporting the use of CCM in ESKD for elderly and frail patients [42], including benefits in quality of life [20,21,22,23,43], and cost reduction [44,45,46], without survival impairment [12,13,14], nephrologists frequently categorize CCM as palliative care [47,48]. Even studies reporting CCM as a successful intervention often label CCM as palliative care, which contributes to ambiguity regarding its appropriate role in ESKD management for older, frail patients. Referring to CCM as palliative care is not merely a semantic issue; it suggests that CCM is an ineffective intervention and prioritizes end-of-life strategies over active treatments that can improve clinical outcomes and prognosis. The present case reports indicate that CCM can be highly effective in older, frail ESKD patients, enhancing both quality of life and survival following hemodialysis failure.

These two case reports also underscore several critical considerations in the management of ESKD. First, they challenge the notion that dialysis, particularly hemodialysis, is the sole reliable treatment option at the end stage of kidney disease. While renal replacement therapy (RRT) has demonstrated survival benefits in the general population, this report does not dispute its value in adult or older patients without significant comorbidities. Instead, it highlights the need to carefully evaluate and balance the risks and benefits of available treatments for each individual to facilitate personalized therapy. Personalized treatment is already recognized as best practice in nephrology, as seen in the preference for kidney transplantation over RRT in eligible patients [49,50,51,52,53,54,55], the selection of peritoneal dialysis for those with poor venous access or severe heart failure [37,56,57,58], and the choice of hemodialysis for patients with prior major abdominal surgery [37]. In elderly and frail patients, whose healthcare needs extend beyond kidney replacement, RRT may not address all clinical challenges and can decrease quality of life and increase hospitalization rates due to complications [24,25]. Therefore, the underlying CKD, assessment of comorbidities, functional independence, the rate of CKD progression, and support from family or caregivers are essential in determining optimal treatment for ESKD [37,59,60,61,62,63]. As the proportion of elderly and frail ESKD patients has increased over the past decade [63], nephrologists increasingly care for patients for whom kidney failure is not the primary determinant of health, and for whom social and economic factors significantly affect survival and quality of life [63,64]. In this context, ethical considerations regarding the most appropriate ESKD treatment should balance individual and community benefits. Pursuing RRT in patients without demonstrable survival or quality-of-life benefit is inadequate for the individual and inefficient for the community, which may lose resources that could benefit others [62].

Secondly, nephrologists and physicians should prioritize patients’ treatment preferences and avoid assuming that a single approach is universally optimal. Elderly patients often have distinct perspectives on what they can manage and may not always prioritize survival as the primary goal in their treatment decisions. However, in this patient population, evidence-based medicine does not provide definitive conclusions about survival outcomes because baseline differences between patients receiving CCM and those undergoing RRT confound comparative studies [65]. Therefore, survival should not be the sole determinant in the nephrologist’s assessment. To support patients unfamiliar with ESKD complications and treatment options, it is reasonable to provide detailed information about potential complications during informed consent, focusing on the scenarios most relevant to their choices. For hemodialysis, patients should be informed about risks such as hypotension and cramps [66,67], thrombosis of vascular access [67,68,69], sepsis [70,71,72], progressive reduction in urine output [73], the need for fluid restriction, potential loss of autonomy, and increased risk of mortality in the first year of dialysis. For CCM, nephrologists should explain the impact of dietary protein restriction on daily habits and the associated risk of sarcopenia and protein-energy wasting [27,28,29,31], the necessity of adherence to pharmacological treatments for ESKD complications, the challenge of maintaining adequate hydration, and the risk of losing diuresis if comorbidities worsen, which can have a severe impact on survival. Effective communication between physicians, patients, and families is essential for shared decision-making [74,75]. However, communication is often hindered by misunderstandings of medical terminology and the complexity of the issues, which may adversely affect decision-making [76,77].

Thirdly, a personalized approach to dietary intervention is essential for several reasons. Considering comorbidities and conducting nutritional assessments are therapeutic objectives that can reduce uremic toxins and enhance patient well-being [78,79,80]. Additionally, individualized dietary planning is critical for improving adherence to dietary restrictions [81,82]. For example, Patient 2 initially adhered to dietary guidelines but later failed to comply, resulting in a significant decline in laboratory results. Dietary adjustments and support from a dietitian improved compliance with a low-protein diet, representing a reasonable compromise for the patient. Achieving dietary adherence is challenging, particularly in chronic diseases where restrictions are long-term or lifelong [83,84]. Therefore, CCM outpatient clinics should function not only as venues for treatment or dietary modification but also as educational settings that provide information about healthy behaviors and reinforce motivation to maintain them [85,86]. Follow-up should be scheduled every 4 to 8 weeks to allow for treatment adjustments, monitor kidney function and nutritional status, and support patients in CCM. While intensive follow-up may be impractical due to economic and staffing constraints [62], CCM remains a preferable option considering the resource burden of dialysis [62]. However, this approach may be limited by patients’ social circumstances. In elderly patients lacking strong social support, regular clinic attendance may be difficult [87,88,89]. Implementing community-based services and telemedicine programs can improve adherence among vulnerable individuals [90,91,92].

Finally, we underscore that in these two cases, the CCM was successful not only in terms of survival but also in achieving the standard of care recommended for ESKD patients [37], with a contextually improved nutritional status. Erythropoietin dose decreased; sodium and potassium balance was achieved; calcium and phosphate control was obtained, especially in patient 2, allowing a reduction in phosphate binders; vitamin D level improved; and albumin and prealbumin improved as well. These are all signs of good adherence to diet and treatment and of good supportive care [93,94,95,96,97], which we consider a basic element in frail patients.

Given the nature of this report, which describes two patients who voluntarily discontinued hemodialysis, certain limitations are inherent. By definition, a case report represents low-level evidence, although the development of evidence-based medicine often begins with individual observations. Furthermore, this therapeutic approach cannot be generalized to all patients; factors such as residual kidney function, frailty, functional status, social context, and experience with the CCM outpatient clinic must be considered. Within these limits, these case reports remain significant for reconsidering the potential role of CCM and the possible limits of dialysis.

## 4. Conclusions

These two case reports highlight the role of CCM in the management of ESKD as a successful treatment, challenging the common view that hemodialysis is the standard of care for ESKD. Furthermore, these case reports also underscore the importance of patients’ determination in treatment management.

## Figures and Tables

**Table 1:** Clinical parameters of patient 1 in the last month of HD and after CCM.

Clinical Parameters	Before HD Discontinuation	3 Months After CCM	1 Year After CCM
Weight (kg)	51	49	52
Urine output (cc/d)	1200	1500	1500
MUST score	2	1	1
Creatinine (umol/L)	//	344	359
Urea (mmol/L)	18.5	16.8	19.9
Sodium (mmol/L)	134	137	139
Potassium (mmol/L)	3.3	4.2	4.9
Calcium (mmol/L)	2.3	2.2	2.33
Phosphate (mmol/L)	1.36	1.52	1.45
PTH (pg/mL)	172	105	165
VitD25OH (mmol/L)	22.5	82.8	56
Bicarbonate (mmol/L)	21.2	25	25.9
Haemoglobin (g/L)	98	100	101
Uric Acid (mmol/L)	636	345	345
Albumin (mmol/L)	28	28	33
Proteinuria (g/d)	0.14	0.13	33
HbA1c (mmol/mol)	47	42	46
Erythropoietin (UI/week)	20,000	7500	10,000
Phosphate binder (g/d)	no	no	no
Potassium binder	no	no	no
Sodium bicarbonate	no	no	no
Furosemide (mg/d)	125	125	125
Spironolactone (mg/d)	25	25	25
Allopurinol dose (mg/d)	50	50	50

Footnotes: CCM Comprehensive Conservative Management, HD Hemodialysis, MUST Malnutrition Universal Screening Tool, PTH parathormone, HbA1c glycate Hemoglobin.

**Table 2:** Clinical parameters of patient 2 before and after CCM.

Clinical Parameters	Before HD Discontinuation	1 Months After CCM	1 Year After CCM
Weight (kg)	46	46	53
Urine output (ml/d)	1400	1500	1300
MUST score	3	1	0
Creatinine (umol/L)	//	405	388
Urea (mmol/L)	25.7	15.9	17.3
Sodium (mmol/L)	138	141	142
Potassium (mmol/L)	3.8	4.2	3.4
Calcium (mmol/L)	2.13	2.2	2.32
Phosphate (mmol/L)	1.65	1.13	1.33
PTH (pg/mL)	145	98	106
VitD25OH (mmol/L)	65	78	92.8
Bicarbonate (mmol/L)	25.4	27.3	28.4
Haemoglobin (g/L)	117	126	109
Uric Acid (mmol/L)	0.25	0.3	0.31
Albumin (mmol/L)	30	36	34
Proteinuria (g/d)	0.3	0.25	0.14
HbA1c (mmol/mol)	47	52	48
Erythropoietin (UI/week)	5000	3300	2500
Phosphate binder (g/d)	4.8 (Sevelamer)	no	no
Potassium binder (g/d)	no	no	no
Sodium bicarbonate (g/d)	no	no	no
Furosemide (mg/d)	100	100	250
Allopurinol (mg/d)	100	100	100

Footnotes: CCM Comprehensive Conservative Management, HD Hemodialysis, MUST Malnutrition Universal Screening Tool, PTH parathormone, HbA1c glycate Hemoglobin.

## Data Availability

Data available on request due to restrictions (e.g., privacy, legal or ethical reasons).

## Abbreviations

The following abbreviations are used in this manuscript:

CCM	Comprehensive conservative management
ESKD	End-stage kidney disease
CKD	Chronic Kidney disease
RRT	Renal Replacement therapy
